# Analysis of the effects of exposure to polychlorinated biphenyls and chlorinated pesticides on serum lipid levels in residents of Anniston, Alabama

**DOI:** 10.1186/1476-069X-12-108

**Published:** 2013-12-11

**Authors:** Zafar Aminov, Richard F Haase, Marian Pavuk, David O Carpenter

**Affiliations:** 1 Institute for Health and the Environment, University at Albany, 5 University Place, Rensselaer, NY 12144, USA; 2 Agency for Toxic Substances and Disease Registry, Atlanta, GA, USA

**Keywords:** Cholesterol, Triglycerides, Persistent organic pollutants, LDL cholesterol, HDL cholesterol, Hexachlorobenzene, DDT, PCBs

## Abstract

**Background:**

Anniston, Alabama, is the site of a former Monsanto plant where polychlorinated biphenyls (PCBs) were manufactured from 1929 until 1971. Residents of Anniston are known to have elevated levels of PCBs. The objective of the study was to test the hypothesis that levels of the various lipid components (total cholesterol, LDL cholesterol, HDL cholesterol, triglycerides) are differentially associated with concentrations of total PCBs and total pesticides, and further that different congeners, congener groups and different pesticides do not have identical associations in serum samples obtained from Anniston residents in a cross-sectional study.

**Methods:**

Fasting serum samples were obtained from 575 residents of Anniston who were not on any lipid-lowering medication and were analyzed for 35 PCB congeners, nine chlorinated pesticides, total cholesterol, LDL and HDL cholesterol and triglyceride concentrations. Associations between toxicant concentrations and lipid levels were determined using multiple linear regression analysis.

**Results:**

We observed that elevated serum concentrations of lipids were associated with elevated serum concentrations of ΣPCBs and summed pesticides in analyses adjusted for age, race, gender, BMI, alcohol consumption, smoking and exercising status. The strongest associations were seen for PCB congeners with three, four, or at least eight substituted chlorines. Mono-ortho substituted congeners 74 and 156, di-ortho congeners 172 and 194, and tri- and tetra-ortho congeners 199, 196–203, 206 and 209 each were significantly associated with total lipids, total cholesterol and triglycerides. Serum concentrations of HCB and chlordane also had strong associations with lipid components.

**Conclusions:**

Increased concentrations of PCBs and organochlorine pesticides are associated with elevations in total serum lipids, total cholesterol and triglycerides, but the patterns are different for different groups of PCBs and different pesticides. These observations show selective effects of different organochlorines on serum concentrations of different groups of lipids. This elevation in concentrations of serum lipids may be the basis for the increased incidence of cardiovascular disease found in persons with elevated exposures to PCBs and chlorinated pesticides.

## Background

There has been suggestive evidence for many years that elevations in levels of some persistent organic pollutants (POPs) are associated with elevated levels of serum lipids. Since elevated serum lipids are a major risk factor for cardiovascular disease [1], this association, if causal, may have significant effects on human health. In 1978 two laboratories reported that feeding rats PCBs and/or DDT resulted in elevation of serum cholesterol [2,3]. Oda et al. [4] reported that feeding rats PCBs resulted in hypercholesterolemia, and suggested that PCB exposure resulted in a stimulation of de novo synthesis of liver lipids. Azaïs-Braesco et al. [5] studied the effects of two different PCB congeners and found that the changes in lipid profiles varied with congener structure. Monkeys fed Aroclor 1254 developed elevated triglycerides, but decreased total cholesterol, HDL and LDL cholesterol [6]. Sanyal et al. [7] reported elevations in lipid synthesis by the liver in DDT-treated monkeys.

In human populations exposed to PCBs, several authors have reported elevations in triglycerides [8,9] and total cholesterol [10-12]. Other studies of occupationally exposed persons noted elevations in rates of cardiovascular deaths [13]. In a Native American population higher PCB levels were associated with elevations of both triglycerides and total cholesterol. The elevated PCB levels were also associated with an elevation in self-reported cardiovascular disease which appeared to be secondary to the elevation in serum lipids [14], although that study was limited by the population size and the self-reported nature of the diagnosis. However, General Electric scientists, while reporting an association between levels of serum PCBs and serum lipids, have argued that the PCB elevation is a consequence, not a cause, of the hyperlipidemia [15,16].

Anniston, Alabama, is the home of one of two facilities in the US operated by Monsanto Chemical Company to produce PCBs; PCBs were made in Anniston from 1929 until 1971. In 2003 ATSDR funded a study of the health of Anniston residents through a consortium of universities. The serum PCB levels in 758 Anniston residents, ages 19–93 years, ranged from 0.1 to 170.4 ng/g (ppb), with a median of 3.2 ng/g [17]. These concentrations can be compared to the statement by ATSDR [18] that the average PCB concentration in persons in the United States that are not unusually exposed is between 0.9 and 1.5 ng/g.

The objective of the current study was to assess the relationship between the serum levels of total PCBs, different groups of congeners and single congeners as well as total chlorinated pesticides and pesticide groups with total serum lipids, serum triglycerides, total cholesterol, LDL and HDL cholesterol among residents of Anniston who were not on lipid-lowering medication.

## Methods

Information about the study population, sampling methods, data collection techniques and laboratory analyses have been described in detail elsewhere [17,19], and therefore are presented only briefly here.

The study was reviewed and approved by the Institutional Review Board (IRB) of the University of Alabama at Birmingham (protocol number X050610004) with concurrence from the University at Albany IRB. Written informed consent was obtained from all participants for use of all questionnaire and analytical data for analysis and publication.

### Study site and population

Stratified randomized sampling, based on residential proximity to the plant, was used to choose households where an adult household member was invited to enroll in the study. A total of 1,110 persons ages 18 years and older were enrolled. Interviews were conducted by trained interviewers. The objective of the interview was to obtain information about the study participant’s age, gender, race/ethnicity, medications, and health related behaviors such as exercise, smoking and alcohol consumption, each treated as a yes or no. Alcohol consumption was defined as the subject having had at least one drink in the past 30 days. Exercise was evaluated as 10 minutes or more of vigorous exercise each day, while smoking was defined as having consumed 100 or more cigarettes over the lifetime. Information on current smoking was also available but didn’t provide qualitatively different results when included in the models.

Fasting blood samples were obtained from 774 persons for analysis of serum PCBs and pesticides, serum lipids and fasting glucose. Twelve study participants were excluded from the analysis for different reasons: PCB results failed quality control/assurance procedures for eight subjects, there was no medication information for one subject, no race/ethnicity information for one subject and height and weight parameters were not available for 2 participants. Because we are evaluating the relationships between exposure to POPs and serum lipids in this report, analysis will be limited to study of the 575 participants who were not taking any kind of lipid-lowering medication. While other medications can also alter lipid levels we did not adjust for any except to exclude individuals specifically on lipid-lowering medication.

### Laboratory analyses

Serum levels of 35 PCB congeners with ortho-substituted chlorines (28, 44, 49, 52, 66, 74, 87, 99, 101, 105, 110, 118, 128, 146, 149, 151, 153, 156, 157, 138–158, 167, 170, 172, 177, 178, 180, 183, 187, 189, 194, 195, 199, 196–203, 206, 209) and 9 organochlorine pesticides [hexachlorobenzene (HCB), β-hexachlorocyclohexane (β-HCCH), γ-HCCH, oxychlordane, transnonachlor, dichlorodiphenyltrichloroethanes (p,p’-DDE, p,p’-DDT, o,p’-DDT) and mirex] were measured by high resolution gas chromatography/isotope-dilution high resolution mass spectrometry at the Centers for Disease Control and Prevention’s National Center for Environmental Health laboratory [20]. At CDC, analyses were conducted as technical assistance and in compliance with Research Determination procedure; an exemption from the CDC IRB was obtained.

Serum levels of different lipid fractions (triglycerides, total cholesterol, LDL cholesterol and HDL cholesterol) were measured at the Clinical Chemistry Laboratory of the Jacksonville, Alabama Medical Center. Total serum lipid levels were calculated using the formula proposed by Phillips et al. [21] and confirmed by Bernert et al. [22].

$$\begin{array}{ll} \mathrm{Total}\;\mathrm{Lipids} & =2.27\;\mathrm{Total}\;\mathrm{Cholesterol}+\mathrm{Triglycerides}\\ & \;+0.623 \end{array}$$

### Statistical analysis

All statistical analyses were conducted using SAS System 9.1.3 package (SAS Institute, Inc., Cary, NC). Descriptive statistics were calculated for outcome variables, demographic characteristics, and exposure variables. Student’s *t*-test was used in order to assess differences of age, serum concentrations of various lipid fractions and POPs. Normality of covariates was assessed graphically and by the Kolmogorov-Smirnov test. Lipid concentrations were somewhat skewed and POPs concentrations were highly skewed. Therefore both were natural log transformed for the regression analysis.

Since PCB congeners and organochlorine pesticides have different molecular structures, they may have different toxicities and health effects [23]. Various groupings based on structure-activity properties/mechanism of action were proposed as recently reviewed by Warner et al. [24]. In human serum samples, all proposed PCB congeners groupings suffer from the common problem of including several PCB congeners that are highly correlated with the sum of PCBs in human sera (r > 0.90; e.g. dioxin-like, potentially anti-estrogenic, immunotoxic congeners, including PCBs 74, 105, 118, 138, 156, 157, 167, and 170), thus providing little or no discrimination when used in statistical analyses. Hence, we evaluated associations between the outcome variables and groups of PCB congeners only as outlined above, i.e. by number of chlorines and number of ortho substituted chlorines) and pesticides, as well as single PCB congeners. For the pesticides we considered total DDTs (the sum of p,p’-DDE, p,p-DDT and o,p’DDT), the sum of the chlordanes (oxychlordane and trans-nonachlor) and the sum of the hexachlorocyclohexanes (β-HCCH and γ-HCCH), as well as HCB and mirex. PCB congeners were grouped first by number of ortho-substituted chlorines and secondly by the number of total chlorines.

Several PCB congeners (52, 49, 44, 101, 110, 151, 149, and 128) and γ-HCCH were excluded from analyses because of high prevalence (more than 60%) of values below the detection limit. For the remaining PCB congeners and pesticides, values below detection limits were replaced by the detection level divided by the square root of 2.

Multiple linear regression modeling was used to evaluate relationships between five outcome variables (serum concentrations of total lipids, total cholesterol, HDL cholesterol, LDL cholesterol, and triglycerides) and exposure variables (serum levels of total and various groups of PCBs and pesticides). Each regression model was adjusted for confounders defined by selected demographic characteristics of the study population. Because of severe multicollinearity among the various groups of POPs, two models were tested. In Model 1 results were adjusted only for age, age quadratic (age^2^), body mass index (BMI, analyzed as a continuous variable), race, gender, alcohol consumption, smoking and exercise. Adjustments have been made in previous studies for these biologically important variables often associated with serum lipids and POPs [15,16,25-28]. In Model 2, additional adjustments were made for all other POPs except the specific one under investigation. Regression coefficients in Model 1 are free of the excessive multicollinearity that exists among individual congeners of PCBs, groups of congeners of PCBs, and individual pesticides. Attempts to evaluate the unique contribution of an individual congener, or a group of congeners, in Model 2 often fail because of excessive collinearity among predictors and lead to the appearance of statistical artifacts (e.g., a change in sign of coefficient from the original correlation) (See Discussion and Additional file 1). Where Model 1 coefficients differ markedly from Model 2 coefficients, the difference is largely due to multicollinearity [29] and indicates that use of Model 2 is not appropriate. However Model 2, when results do not differ markedly from those obtained in Model 1, allows the possibility of identification of which of the POPs under study is primarily responsible for the association, and is theoretically superior.

Predictive models were validated by assessing R-square statistics and by testing the models in sub-samples of the study population. For main outcomes, results were considered significant at p < 0.05. When each outcome variable was regressed with 27 PCB congeners separately [or in PCB congener groups based on number of chlorines], results were considered significant at p < 0.0019 (0.05/27) [30].

Spearman’s correlation coefficients were calculated for groups of PCBs by total number of chlorines on the molecule, the number of ortho-substituted chlorines on the molecule, by pesticides groups as well as for individual PCB congeners.

## Results

Table 1 shows demographic characteristics of the study population. All essential data were available for 575 study participants, none of whom were on any lipid-lowering medication that might obscure the results, although smoking and physical activity data were missing for a few subjects. The mean age was 52.2 and the median was 52. More than seventy percent were within the age range of 37–74 years. Those under 36 and older than 75 were 18.43% and 9.04% respectively, and graphically variable age was considered as normally distributed. Seventy percent of subjects were female. There were approximately equal numbers of Caucasians and African Americans. Nearly 70% of subjects did not drink alcohol (defined as at least one drink in the past 30 days), while nearly half smoked (defined as having smoked more than 100 cigarettes over the lifetime) and had regular exercise (defined as having daily moderate physical activity of at least 10 min duration).

**Table 1:** Demographic characteristics of study participants (n = 575)

Age	
*18 – 36*	106 (18.43%)
*37 – 55*	225 (39.13%)
*56 – 74*	192 (33.39%)
*75 – 93*	52 (9.04%)
Gender	
*Female*	407 (70.78%)
*Male*	168 (29.22%)
Race	
*Caucasian-American*	288 (50.09%)
* African-American*	287 (49.91%)
Alcohol consumption	
*Yes*	177 (30.78%)
*No*	398 (69.22%)
Smoking	
*Yes*	304* (53.05%)
*No*	269* (46.95%)
Physical activity	
*Yes*	319** (55.87%)
*No*	252**(44.13%)

*n = 573.

**n = 571.

Table 2 shows information on BMI and analytical values for lipids and contaminants for the study population. The mean BMI of 30.88 kg/m^2^, with standard deviation of 8.03, indicates a high rate of obesity. Mean serum concentrations of total cholesterol, LDL cholesterol, HDL cholesterol and triglycerides were within “normal” ranges (triglycerides <200 mg/dL; total cholesterol, 120–200 mg/dL; HDL cholesterol, 35–86 mg/dL; LDL cholesterol, 80–130 mg/dL) [31], although values both lower and higher were found in some individuals. The range of exposures was wide, with serum PCB concentrations ranging from 0.1 to 170.4 ppb. Total pesticide concentrations ranged from 0.15 to 39.07 ppb wet weight. When PCB congeners were grouped by numbers of ortho chlorines or total chlorines, the highest concentration was observed for di-ortho PCBs (mean concentration, 3.95 ppb) and for hexachloro PCBs (mean concentration, 2.59 ppb). Regarding organochlorine pesticides, DDT and its derivatives composed almost 85% of mean total concentrations of pesticides. The other pesticides were in much lower concentrations.

**Table 2:** Lipid components, body mass index (BMI), and serum concentrations of total PCBs and chlorinated pesticides (n = 575)

	**Mean**	**Median**	**Standard deviation**	**Min**	**Max**
BMI (kg/m^2^)	30.88	30.00	8.03	16.00	65.00
Total Lipids (mg/dL)	636.88 (mg/dL)	609.80 (mg/dL)	153.07 (mg/dL)	335.80 (mg/dL)	1436.20 (mg/dL)
Total Cholesterol (mg/dL)	195.85 (mg/dL)	192.00 (mg/dL)	42.76 (mg/dL)	84.00 (mg/dL)	373.00 (mg/dL)
HDL (mg/dL)	48.53 (mg/dL)	46.00 (mg/dL)	15.89 (mg/dL)	19.00 (mg/dL)	148.00 (mg/dL)
LDL (mg/dL)	121.68 (mg/dL)	119.00 (mg/dL)	37.19 (mg/dL)	36.00 (mg/dL)	300.00 (mg/dL)
Triglycerides (mg/dL)	130.14 (mg/dL)	106.00 (mg/dL)	92.13 (mg/dL)	15.00 (mg/dL)	929.00 (mg/dL)
Total PCBs (ng/g ww)	6.33	3.16	12.61	0.11	170.42
Mono-ortho congeners (ng/g ww)	0.85	0.37	2.09	0.02	34.28
Di-ortho congeners (ng/g ww)	3.95	1.95	8.05	0.06	107.95
Tri- and tetra-ortho congeners (ng/g ww)	1.53	0.72	2.84	0.02	28.19
Tri- and tetrachloro congeners (ng/g ww)	0.16	0.09	0.30	0.01	4.33
Pentachloro congeners (ng/g ww)	0.68	0.23	1.89	0.02	34.40
Hexachloro congeners (ng/g ww)	2.59	1.17	5.58	0.04	77.70
Heptachloro congeners (ng/g ww)	1.81	0.89	3.56	0.02	41.46
Octa-, nona- and decachloro congeners (ng/g ww)	1.10	0.51	2.11	0.01	21.65
Total Pesticides (ng/g ww)	4.31	2.56	5.48	0.15	39.07
*pp-DDE*, *op-DDT*, *pp-DDT* (ng/g ww)	3.65	1.87	5.03	0.03	38.51
Oxychlordane, t-nonahlordane (ng/g ww)	0.47	0.31	0.68	0.02	10.44
Mirex (ng/g ww)	0.10	0.06	0.19	0.00	2.67
HCB (ng/g ww)	0.07	0.06	0.07	0.02	1.48
β-HCCH, γ-HCCH (ng/g ww)	0.12	0.06	0.17	0.01	1.77

Table 3 presents results of multiple linear regression models of association between five outcome variables and serum total concentrations of both PCBs and pesticides. Separate regression analyses were fitted for each outcome variable. Results of analyses for total PCBs and total pesticides were considered significant at p < 0.05. Each outcome variable was regressed on PCBs and pesticides with additional adjustment for the confounders (age, gender, race, BMI, alcohol consumption, smoking and exercise) (Model 1). Race and age were significant covariates in both Models 1 and 2, but gender, alcohol consumption, smoking and exercise were not. Both total PCBs and total pesticides were significantly and positively associated with elevations in total lipids, total cholesterol and triglycerides, but not with HDL or LDL cholesterol. In order to evaluate the unique effect of PCBs we therefore adjusted for total pesticides as well as the other confounders, and adjusted results for total pesticides for total PCBs as well as other confounders (Model 2). Total PCBs were significantly and positively associated with total lipids, while total pesticides were significantly and positively associated with levels of total lipids, total cholesterol and triglycerides. There was no significant association between HDL or LDL cholesterol with either total PCBs or total pesticides. The relationship between serum total POPs (the sum of total PCBs plus total pesticides) with each of five outcome variables is presented in Additional file 2. When total PCB concentration was adjusted for total pesticides (Model 2), the significant relationship with total cholesterol and triglycerides disappeared. In contrast, when total pesticides was adjusted for total PCBs, the significant associations with total lipids, total cholesterol and triglycerides, but not LDL or HDL cholesterol, did not change markedly from results of Model 1.

**Table 3:** Results of multiple linear regression analysis of association between natural log transformed serum concentrations of PCBs and chlorinated pesticides and lipid fractions (n = 575)

		**Total PCBs**		**Total pesticides**	
		**Model 1**	**Model 2**	**Model 1**	**Model 2**
	β	0.05	0.03	0.07	0.06
Total lipids	p-value	<0.0001	0.0433	<0.0001	0.0001
	SP R^2^2	0.0280	0.0063	0.0447	0.0230
	β	0.03	0.02	0.04	0.03
Total Cholesterol	p-value	0.0090	0.1816	0.0017	0.0277
	SP R^2^2	0.0112	0.0029	0.0162	0.0079
	β	0.00	0.01	−0.01	−0.01
HDL cholesterol	p-value	0.9685	0.7388	0.5714	0.5122
	SP R^2^2	0.0000	0.0002	0.0005	0.0002
	β	0.03	0.02	0.03	0.02
LDL cholesterol	p-value	0.1233	0.3382	0.1209	0.3301
	SP R^2^2	0.0041	0.0016	0.0041	0.0016
	β	0.10	0.05	0.15	0.12
Triglycerides	p-value	0.0007	0.1365	<0.0001	0.0007
	SP R^2^2	0.0178	0.0034	0.0321	0.0177

Multiple linear regression models to assess the associations between serum total concentrations of PCBs and pesticides with serum total concentrations of lipids and its fractions after adjustment.

Model 1: The estimates are adjusted for age (as well as age quadratic), race, gender, BMI, alcohol consumption, smoking and exercising status, but results for total PCBs are not adjusted for total pesticides and results for total pesticides are not adjusted for total PCBs.

Model 2: The estimates are adjusted for age (as well as age quadratic), race, gender, BMI, alcohol consumption, smoking and exercising status, but with additional adjustment for total PCBs for the pesticide results and for total pesticides for the PCB results.

SP R^2^2 – Type II semi-partial R^2^.

Table 4 shows results of the associations between serum lipid concentrations with concentrations of PCB congeners classified by number of ortho chlorines. Each outcome variable was regressed with 27 PCB congeners categorized into 3 groups based on number of ortho chlorines. Results were considered significant at p < 0.0019 (0.05/27) [30]. Separate results are presented with adjustment only for confounders other than the contaminants (Model 1) and with additional adjustment for all other groups of POPs (total pesticides and both of the other PCB groups) (Model 2). With Model 1 there were significant positive associations between concentrations of mono-ortho congeners and total lipids, di-ortho congeners with total lipids and triglycerides, and tri-plus tetra-ortho congeners with total lipid, triglycerides and total cholesterol. No association was observed with HDL or LDL cholesterol. When adjusted for other POPs (Model 2), only tri- plus tetra-ortho PCBs showed statistically significant positive correlations with total lipids and triglycerides.

**Table 4:** Results of multiple linear regression analysis of the associations between natural log transformed serum concentrations of groups of PCB congeners differing by the number of ortho- substituted chlorines and the various lipid fractions (n = 575)

		**Mono-ortho**		**Di-ortho**		**Tri-/Tetra-ortho**	
		**Model 1**	**Model 2**	**Model 1**	**Model 2**	**Model 1**	**Model 2**
Total lipids	β	0.04	−0.05	0.05	−0.09	0.06	0.12
	p-value	0.0005	0.0814	<0.0001	0.0433	<0.0001	<0.0001
	SP R^2^2	0.0193	0.0040	0.0245	0.0053	0.0391	0.0209
Total Cholesterol	β	0.03	−0.02	0.03	−0.08	0.04	0.09
	p-value	0.0262	0.5254	0.0169	0.0846	0.0013	0.0047
	SP R^2^2	0.0081	0.0001	0.0094	0.0044	0.0169	0.0120
HDL cholesterol	β	0.01	0.08	−0.00	−0.05	−0.00	−0.02
	p-value	0.4722	0.0297	0.9741	0.4145	0.8036	0.6888
	SP R^2^2	0.0008	0.0072	0.0000	0.0010	0.0001	0.0010
LDL cholesterol	β	0.02	−0.03	0.02	−0.09	0.04	0.11
	p-value	0.2997	0.4565	0.1728	0.2056	0.0285	0.0133
	SP R^2^2	0.0018	0.0009	0.0032	0.0026	0.0082	0.0099
Triglycerides	β	0.08	−0.16	0.09	−0.20	0.12	0.31
	p-value	0.0117	0.0219	0.0018	0.0754	<0.0001	<0.0001
	SP R^2^2	0.0099	0.0069	0.0152	0.0042	0.0279	0.0211

Model 1: The estimates are adjusted for age (as well as age quadratic), race, gender, BMI, alcohol consumption, smoking and exercising status, but *not* adjusted for the two remaining PCB groups and chlorinated pesticides.

Model 2: The estimates are adjusted for age (as well as age quadratic), race, gender, BMI, alcohol consumption, smoking and exercising status, and adjusted for the remaining two PCB groups and chlorinated pesticides.

SP R^2^2 – Type II semi-partial R^2^.

Among the results shown in Table 4, there are 10 separate instances in which a reversal of signs of the regression coefficients from positive to a negative occurs between the analyses of Model 1 and Model 2. We call the reader’s attention to the fact that these results should be treated most cautiously, as they are the result of suppression effects due to severe multicollinearity among the predictors. We give further details of these statistical artifacts in the Additional file 1.

The relationships between serum lipids with PCB congeners grouped by numbers of total chlorines are shown in Table 5. Each outcome variable was regressed with 27 PCB congeners categorized into 5 groups based on number of chlorines. Results were considered significant at p < 0.0019 (0.05/27) [30]. Prior to adjustment for other POPs (Model 1), all groups showed significant associations with total lipids and triglycerides. Associations with total cholesterol were observed for groups with three or four, six, seven, and eight or more chlorines. The only significant association with LDL cholesterol was with eight or more chlorines, and there were no associations with HDL cholesterol. These associations disappeared in Model 2 except for the significant positive association between total lipids and PCBs with three or four chlorines.

**Table 5:** Results of multiple linear regression analysis of association between natural log transformed serum concentrations of groups of PCB congeners based on the number of total chlorines and the various lipid fractions (n = 575)

		**Tri- and tetra chloro**		**Penta-chloro**		**Hexa-chloro**		**Hepta-chloro**		**Octa-, nona-, and deca-chloro**	
		**Model 1**	**Model 2**	**Model 1**	**Model 2**	**Model 1**	**Model 2**	**Model 1**	**Model 2**	**Model 1**	**Model 2**
Total lipids	β	0.06	0.08	0.03	−0.06	0.04	0.02	0.05	−0.04	0.06	0.08
	p-value	<0.0001	0.0008	0.0081	0.0653	0.0004	0.7883	<0.0001	0.5873	<0.0001	0.0055
	SP R^2^2	0.0318	0.0172	0.0114	0.0052	0.0199	0.0001	0.0302	0.0004	0.0480	0.0118
Total cholesterol	β	0.04	0.07	0.02	−0.06	0.02	0.04	0.03	−0.06	0.04	0.07
	p-value	0.0019	0.0041	0.1332	0.0715	0.0308	0.5659	0.0063	0.4051	0.0002	0.0229
	SP R^2^2	0.0157	0.0132	0.0037	0.0052	0.0077	0.0005	0.0122	0.0011	0.0224	0.0083
HDL cholesterol	β	0.01	0.02	0.06	0.02	0.00	−0.08	0.00	0.09	−0.01	−0.05
	p-value	0.5381	0.6441	0.6625	0.5902	0.9262	0.3807	0.9658	0.3222	0.6741	0.2344
	SP R^2^2	0.0006	0.0003	0.0002	0.0004	0.0000	0.0012	0.0000	0.0015	0.0003	0.0021
LDL cholesterol	β	0.03	0.06	0.01	−0.08	0.02	0.11	0.03	−0.15	0.04	0.11
	p-value	0.1333	0.1304	0.5504	0.1407	0.2239	0.2837	0.112	0.1432	0.0080	0.1432
	SP R^2^2	0.0039	0.0039	0.0006	0.0037	0.0025	0.0019	0.0043	0.0036	0.0060	0.0110
Triglycerides	β	0.11	0.14	0.05	−0.07	0.08	−0.11	0.11	0.02	0.14	0.19
	p-value	0.0008	0.0284	0.0458	0.4191	0.0077	0.5383	0.0004	0.9287	<0.0001	0.0130
	SP R^2^2	0.0174	0.0072	0.0062	0.0010	0.0111	0.0006	0.0197	0.0000	0.0350	0.0093

Model 1: The estimates are adjusted for age (as well as age quadratic), race, gender, BMI, alcohol consumption, smoking and exercising status, but *not* adjusted for the remaining PCB groups and chlorinated pesticides.

Model 2: The estimates are adjusted for age (as well as age quadratic), race, gender, BMI, alcohol consumption, smoking and exercising status, and adjusted for the all remaining PCB groups and chlorinated pesticides.

SP R^2^2 – Type II semi-partial R^2^.

Table 6 provides the associations obtained with single PCB congeners without adjustment for other POPs (Model 1). Each outcome variable was regressed with 27 separate PCB congeners. Results were considered significant at p < 0.0019 (0.05/27) [30]. The mono-ortho substituted congeners 74 and 156 were significantly associated with total lipids, total cholesterol and triglycerides, as were the higher chlorinated di-ortho congeners 172 and 194, as well as the tri-and tetra-ortho congeners 199, 196–203, 206 and 209. Associations with total lipids and triglycerides only were observed for mono-ortho congeners 28 and 189, di-ortho congeners 170 and 180, and tri-ortho congeners 178, 187, and 195. Mono-ortho congener 157, di-ortho congeners 146 and 153, and tri-ortho congeners 177 and 183 were only associated with total lipids. Mono-ortho congeners 66, 118, 105, and 167 and di-ortho congeners 87, 99, and 138–158 were not associated with any lipids. Only PCBs 194 and 209 had positive associations with LDL but not HDL cholesterol at alpha <0.0019. Application of Model 2 to single congeners was not feasible due to extreme multicollinearity.

**Table 6:** Results of multiple linear regression analysis of associations between natural log transformed serum concentrations of individual PCB congeners and various lipid fractions (n = 575)

			**Total lipids**	**Total cholesterol**	**Triglycerides**	**HDL cholesterol**	**LDL cholesterol**
Mono-ortho PCBs	PCB28	β	0.04	0.03	0.10	0.02	0.01
		p-value	<0.0001	0.0125	0.0007	0.2387	0.5784
	PCB66	β	0.03	0.02	0.06	0.03	0.00
		p-value	0.0037	0.0504	0.0127	0.0195	0.9542
	PCB74	β	0.06	0.04	0.10	0.00	0.03
		p-value	<0.0001	0.0017	0.0013	0.8356	0.0784
	PCB105	β	0.03	0.02	0.05	0.01	0.01
		p-value	0.0069	0.1010	0.0418	0.2926	0.6506
	PCB118	β	0.02	0.01	0.04	0.01	0.01
		p-value	0.0169	0.1880	0.0725	0.5591	0.6363
	PCB156	β	0.06	0.04	0.11	−0.00	0.04
		p-value	<0.0001	0.0009	0.0003	0.9040	0.0243
	PCB157	β	0.04	0.03	0.07	0.01	0.03
		p-value	0.0005	0.0116	0.0268	0.4697	0.1247
	PCB167	β	0.02	0.01	0.03	0.01	0.01
		p-value	0.0916	0.4734	0.3205	0.4060	0.7185
	PCB189	β	0.05	0.03	0.10	0.01	0.02
		p-value	<0.0001	0.0212	0.0012	0.4239	0.2496
Di-ortho PCBs	PCB87	β	0.02	0.02	0.01	0.02	0.02
		p-value	0.1609	0.0780	0.8130	0.1054	0.2503
	PCB99	β	0.03	0.02	0.05	−0.00	0.01
		p-value	0.0031	0.0743	0.0281	0.8366	0.3161
	PCB146	β	0.03	0.02	0.06	−0.00	0.01
		p-value	0.0019	0.0949	0.0083	0.7605	0.3149
	PCB153	β	0.04	0.02	0.07	0.00	0.02
		p-value	0.0004	0.0276	0.0079	0.9119	0.1978
	PCB138-158	β	0.03	0.02	0.06	−0.00	0.01
		p-value	0.0029	0.0921	0.0193	0.7824	0.3898
	PCB170	β	0.06	0.04	0.13	−0.00	0.03
		p-value	<0.0001	0.0021	<0.0001	0.9292	0.0800
	PCB172	β	0.06	0.04	0.12	0.01	0.03
		p-value	<0.0001	0.0004	<0.0001	0.6173	0.0402
	PCB180	β	0.05	0.03	0.11	−0.00	0.03
		p-value	<0.0001	0.0083	0.0005	0.9808	0.1221
	PCB194	β	0.06	0.04	0.11	−0.01	0.05
		p-value	<0.0001	<0.0001	<0.0001	0.6830	0.0019
Tri- and tetra-ortho PCBs	PCB177	β	0.04	0.02	0.08	−0.01	0.02
		p-value	0.0003	0.0345	0.0026	0.6422	0.2031
	PCB178	β	0.04	0.03	0.08	0.00	0.03
		p-value	<0.0001	0.0074	0.0009	0.9988	0.0463
	PCB183	β	0.04	0.02	0.07	−0.00	0.03
		p-value	0.0004	0.0175	0.0101	0.8584	0.0966
	PCB187	β	0.04	0.03	0.08	0.00	0.02
		p-value	<0.0001	0.0106	0.0010	0.7736	0.1418
	PCB195	β	0.05	0.03	0.12	0.00	0.03
		p-value	<0.0001	0.0061	<0.0001	0.9093	0.0955
	PCB199	β	0.05	0.03	0.11	0.00	0.03
		p-value	<0.0001	0.0010	<0.0001	0.9232	0.0314
	PCB196-203	β	0.06	0.04	0.13	−0.00	0.04
		p-value	<0.0001	0.0005	<0.0001	0.8600	0.0295
	PCB206	β	0.06	0.04	0.13	−0.01	0.05
		p-value	<0.0001	0.0002	<0.0001	0.4059	0.0030
	PCB209	β	0.05	0.04	0.11	−0.01	0.05
		p-value	<0.0001	0.0001	<0.0001	0.5693	0.0017

The estimates are adjusted only for age (as well as age quadratic), gender, BMI, alcohol consumption, smoking and exercising status. The estimate for each PCB congener is *not* adjusted for the remaining PCB congeners nor for pesticides.

A similar analysis for the groups of pesticides is shown in Table 7. Results were considered significant at p < 0.0019. All of the pesticide groups showed a positive association with total lipids in Model 1. In addition, DDT was positively associated with triglycerides, while total chlordane, HCB, and HCCH were positively associated with total cholesterol and triglycerides, with HCB also associated with LDL cholesterol (at alpha < 0.0019). When adjusted for other POPs (total PCBs and all other pesticides) (Model 2), most associations of lipid levels with total DDT and other pesticides disappeared. However, total chlordane remained significantly associated with triglycerides, and all associations from Model 1 for HCB remained unchanged, showing strong positive associations with all lipids except for a non-significant negative association with HDL-cholesterol.

**Table 7:** Results of multiple linear regression analysis of the associations between natural log transformed serum concentrations of the various pesticides or pesticide groups and the various lipid fractions (n = 575)

		**DDT**		**Chlordane**		**Mirex**		**HCB**		**HCCH**	
		**Model 1**	**Model 2**	**Model 1**	**Model 2**	**Model 1**	**Model 2**	**Model 1**	**Model 2**	**Model 1**	**Model 2**
Total lipids	β	0.05	0.00	0.11	0.05	0.05	0.02	0.21	0.21	0.07	−0.03
	p-value	<0.0001	0.8109	<0.0001	0.0037	0.0002	0.2608	<0.0001	<0.0001	<0.0001	0.1205
	SP R^2^2	0.0300	0.0000	0.0875	0.0110	0.0226	0.0016	0.1539	0.0805	0.0438	0.0031
Total Cholesterol	β	0.03	0.00	0.06	0.02	0.03	0.01	0.15	0.18	0.04	−0.04
	p-value	0.0124	0.9873	<0.0001	0.3877	0.0125	0.3697	<0.0001	<0.0001	0.0018	0.0629
	SP R^2^2	0.0103	0.0000	0.0313	0.0011	0.0102	0.0012	0.0880	0.0653	0.0160	0.0052
HDL cholesterol	β	−0.01	0.00	−0.03	−0.03	0.01	0.02	−0.06	−0.08	−0.01	0.02
	p-value	0.5831	0.9323	0.1309	0.3034	0.7112	0.4401	0.0205	0.0267	0.4439	0.5214
	SP R^2^2	0.0005	0.0000	0.0035	0.0016	0.0002	0.0009	0.0080	0.0074	0.0009	0.0006
LDL cholesterol	β	0.02	0.00	0.05	−0.00	0.02	0.00	0.17	0.25	0.03	−0.07
	p-value	0.2229	0.9100	0.0266	0.9531	0.2846	0.8392	<0.0001	<0.0001	0.1862	0.0198
	SP R^2^2	0.0026	0.0000	0.0085	0.0000	0.0020	0.0001	0.0525	0.0577	0.0030	0.0088
Triglycerides	β	0.10	−0.01	0.26	0.18	0.09	0.01	0.44	0.39	0.18	−0.02
	p-value	0.0003	0.8396	<0.0001	<0.0001	0.0054	0.8391	<0.0001	<0.0001	<0.0001	0.6926
	SP R^2^2	0.0206	0.0000	0.0833	0.0212	0.0121	0.0001	0.1059	0.0422	0.0391	0.0002

Model 1: The estimates are adjusted for age (as well as age quadratic), race, gender, BMI, alcohol consumption, smoking and exercising status, but *not* adjusted for the remaining pesticides and PCBs.

Model 2: The estimates are adjusted for age (as well as age quadratic), race, gender, BMI, alcohol consumption, smoking and exercising status, *and* adjusted for all remaining pesticides and PCBs.

SP R^2^2 – Type II semi-partial R^2^.

Additional file 3 presents Pearson correlation coefficients for all of the groups of PCBs and pesticides, and demonstrates the high degree of multicollinearity among some of the groups. Additional file 4 presents Pearson correlation coefficients among the single PCB congeners. Note that the correlation coefficients are greater for higher than lower chlorinated congeners.

## Discussion

We observed that elevated serum concentrations of lipids were significantly associated with serum concentrations of ΣPCBs and summed pesticides in analyses adjusted for age, race, gender, BMI, alcohol consumption, smoking and exercising status. The strongest associations were seen for PCB congeners with three or four ortho and/or at least eight substituted chlorines. Serum concentrations of HCB and chlordane also had strong associations with lipid components. The results reported here are consistent with conclusions made in study of a Native American population where PCB and pesticide levels were found to be positively correlated with serum total cholesterol and triglyceride concentrations (14).

Elevated concentrations of serum cholesterol and triglycerides and low concentrations of HDL cholesterol are major risk factors for cardiovascular disease [1,32]. There is supportive evidence from both human [25,28,33] and animal [3,34,35] studies that exposure to POPs increases risk of cardiovascular disease.

While numerous studies examined association of PCBs with serum lipids as part of other analyses [9,15,16,25,26], the only previous study that has systematically investigated individual concentrations of POPs in relation to levels of serum lipids is that of Lee et al. (27). They studied a population of 90 individuals over 20 years of age, enriched with overweight and obese persons, and reported associations between concentrations of eight chlorinated pesticides and 22 PCB congeners with serum triglyceride and HDL cholesterol levels. They found heterogeneous relationships between serum concentrations of toxicants and lipids. As in our study they report significant association between concentrations of HCB and serum triglyceride levels. Some of the associations for other POPs they report were non-linear when the POPs were presented in quartiles. There are, however, significant differences in some of their results and ours. This is particularly so in relation to the effects of DDE, which they report to be associated with an elevation in triglycerides and suppression of HDL. We did not detect any effect of total DDTs, including DDE, after adjustment for other POPs and we found no significant effect on HDL cholesterol by DDE. The reason for this difference is uncertain, but may reflect the differences in the populations being studied and/or differences in adjustment for other POPs.

Obesity and change in weight also have profound effects on concentrations of lipids and levels of organochlorines. Lind et al. [36] studied life-time weight change in relation of concentration of POPs in the PIVUS study. The average estimated weight change over 50 years was 14.4 kg. Both the sum of OC pesticide concentrations (4.3 kg more weight gain in quintile 5 vs. quintile 1, p < 0.0001) and the sum of the less-chlorinated PCBs were positively related to the estimated weight change (3.7 kg more weight gain in quintile 2 vs. quintile 1, non-linear relationship p = 0.0015). In contrast, the sum of concentrations of highly-chlorinated PCBs were inversely related to estimated weight change (8.4 kg less weight gain in quintile 5 vs. quintile 1, p < 0.0001). Differences in mode of action, toxicokinetics, non-linear relationships and reverse causation might explain these discrepancies [36]. In the analysis of abdominal obesity of this cohort [37], twenty-one plasma POPs (16 polychlorinated biphenyl (PCB) congeners, 3 organochlorine (OC) pesticides, 1 brominated diphenyl ether (BDE), and 1 dioxin) were measured at baseline in 970 participants aged 70 years with prospective analyses in 511 participants re-examined after 5 years. Abdominal obesity was defined by an increased waist circumference. In the cross-sectional analyses, concentrations of the less chlorinated PCBs, OC pesticides such as p,p’-DDE and dioxin had adjusted odds ratios of 2 to 3 for abdominal obesity. Many relations had inverted U-shapes rather than being linear, particularly in women. In contrast, concentrations of highly chlorinated PCBs were strongly inversely associated with abdominal obesity. In a single model including summary measures of the less chlorinated PCBs, highly chlorinated PCBs, and OC pesticides, both the positive associations and inverse associations strengthened. Similar but somewhat weaker associations were seen between POPs and risk of development of abdominal obesity in the prospective analyses.

In addition, experimental animal studies have shown dose-dependent increases in serum levels of total cholesterol and triglycerides in response to exposure to PCBs [3,4,6,38]. Exposure to PCBs changes the patterns of lipid metabolism and synthesis [39]. In this study, animals were fed either an olive oil or a corn-oil rich diet, and then exposed to PCB 77, a coplanar, dioxin-like congener. They found that the PCB treatment in both groups caused induction of genes involved in fatty acid degradation but that there was upregulation of different genes. These observations raise another possible complication, in that while different PCB congeners and different pesticides may induce different genes, different kinds of dietary fat may influence which genes are induced. As suggested above, PCBs can initiate inflammatory responses in vivo, and this inflammation can be either exacerbated or ameliorated by nutrition. Petriello et al. [40] also showed that diets high in certain dietary lipids such as omega-6 fatty acids can worsen PCB-induced vascular toxicity while diets enriched with bioactive food components such as polyphenols and omega-3 polyunsaturated fatty acids can improve the toxicant-induced inflammation. There is evidence that bioactive nutrients protect through multiple cell signaling pathways, but they have shown that lipid raft caveolae and the antioxidant defense controller nuclear factor (erythroid-derived 2)-like 2 (Nrf2) both play a predominant role in nutritional modulation of PCB-induced vascular toxicity. Interestingly, there appears to be an intimate cross-talk between caveolae-related proteins and cellular Nrf2, and focusing on the use of specific bioactive food components that simultaneously alter both pathways may produce a more effective and efficient cytoprotective response to toxicant exposure. The use of nutrition as a protective tool is an economically beneficial means to address the toxicity of persistent environmental toxicants and may become a sensible means to protect human populations from PCB-induced vascular inflammation and associated chronic diseases [40].

The precise mechanisms responsible for the associations between serum POPs and the various serum lipid components are uncertain. Organochlorines are difficult to metabolize, and exposure causes induction of various degradative enzymes, particularly different cytochrome P450s [41,42]. In addition, DNA methylation levels in humans have been found to be altered by several persistent organic pollutants, including PCBs and chlorinated pesticides [43], indicating that these chemicals can also induce epigenetic changes. Liver size in animals actually doubles in situations of high exposure, reflecting enzyme induction [44]. Others have documented specific alternations of enzymes involved in lipid synthesis caused by exposure to single PCB congeners [45]. A variety of genes are either up-or down-regulated by different PCB congeners [46,47], p,p’-DDE [48] and by a mixture of 28 persistent organic pollutants [49]. Thus, there are multiple possible pathways that might lead to selective alteration in rates of synthesis or metabolism of some classes of serum lipids.

### Strengths and weaknesses

This study has the strength of relatively large numbers and approximately equal numbers of African-Americans and Caucasians. Exposure status was evaluated objectively and comprehensively. We have excluded individuals on lipid-lowering medication, which would results in changes in lipid levels. We have adjusted results for the important confounders of age, gender, race, BMI, alcohol consumption, smoking and exercise and have also presented results after adjustment for concentrations of other POPs.

However, this study also has some major limitations. The cross-sectional design does not allow for assessing causality. We have a single point in time measurement of both POPs and lipid levels. There may remain some confounding due to the fact that POPs are found in the lipid layers and migrate together. The associations after adjustment for other POPs, seen primarily only with more highly chlorinated PCB congeners which are more lipophilic, could possibly reflect reverse causality. This explanation is less likely in light of results for chlorinated pesticides (especially HCB). We cannot eliminate the possibility that some other lipophilic chemical(s) that was not measured but migrates together with PCBs and chlorinated pesticides has the dominant action.

All of these POPs reside in the lipid fraction in blood, and we assume that blood lipids are in equilibrium with body fat deposits in fasting individuals [21].

Our results are all based on wet weight measurements of levels of POPs, which are unadjusted for lipids. Whether adjusted directly by the methods of Phillips et al. [21] or indirectly – by incorporating unadjusted lipid values as covariates – as proposed by Schisterman et al. [50], adjustments for lipids are appropriate only for non-lipid dependent variables. Since lipids are the outcome variables of the present study, adjusting them directly or indirectly would effectively remove the covariance of the PCBs and the lipid dependent variables. In our previous studies, in which lipids were not dependent variables, we documented that use of wet weight values adjusted for directly or indirectly yielded very similar results [17,19]. There is increasing evidence for non-linear associations between levels of POPs and some disease outcomes, including effects on serum lipid levels [26,27], and these may affect our study as well. It is well recognized that multicollinearity, the extent to which predictor variables in a multiple regression analysis share common variance, can have seriously debilitating effects on the analysis and its interpretation, and this factor seriously limits our ability to determine which of the PCB congeners and different pesticides, if any, are responsible for the associations we observe (see Additional file 1). Finally, although we excluded all individuals who were on lipid-lowering medication from our study, there is some evidence that some other medications may also lower lipid levels. We also did not have detailed dietary information to estimate total fat intake or type of fat consumed to stratify or adjust in the analyses.

## Conclusions

Serum concentrations of total PCBs (sum of 35 congeners) and total chlorinated pesticides (sum of nine pesticides) were significantly associated with elevated serum levels of total lipids, total cholesterol and triglycerides but not levels of LDL and HDL after adjustment for age, gender, race, BMI, smoking and exercise in a study population who were not on lipid-lowering medication. After adjustment for confounders, associations between different groups of POPs and serum lipids were not homogenous. The PCBs with the strongest associations were those with three, four, or eight or more total chlorines, while, of the pesticides, the most striking associations were observed for hexachlorobenzene, even though concentrations of it were low. Chlordane also was found to be positively associated with some lipid components after adjustment for other contaminant concentrations. These results suggest that, in spite of the fact that concentrations of all of these lipophilic chlorinated compounds are highly correlated, the effects on lipid levels are different for different pesticides and PCB congeners. Our results are consistent with the hypothesis that higher levels of POPs, especially higher chlorinated PCBs and some pesticides, result in elevations of serum lipids and that risk of cardiovascular disease and obesity after exposure to PCBs and chlorinated pesticides is, at least in part, a consequence of the elevations in serum lipids that result from exposure.

## Abbreviations

ATSDR: Agency for toxic substances and disease registry; BMI: Body-mass index; DDE: Dichloro-dichlorophenyl-ethylene; DDT: Dichloro-diphenyl-trichloroethane; DNA: Deoxyribonucleic acid; HCB: Hexachlorobenzene; HCCH: Hexachlorocyclohexane; HDL: High density lipoprotein cholesterol; LDL: Low density lipoprotein cholesterol; PCBs: Polychlorinated biphenyls; POPs: Persistant organic pollutants; Ppb: Parts per billion; Ww: Wet weight.

## Competing interests

DOC has served as an expert witness in legal actions against Monsanto relating to exposure of resident of Anniston, Alabama to PCBs, with all compensation paid to the Research Foundation at the University at Albany. The other authors declare that they have no competing interests.

## Authors’ contributions

ZA participated in the planning of the study, carried out most of the data analysis and this study constituted a part of his PhD thesis. RH reviewed and advised on the statistical analysis and wrote the material contained in the Additional file 1. MP reviewed and advised on all aspects of the study. DOC designed the study and supervised all aspects of data analysis and preparation of the manuscript in addition to receiving the funding for the project. All authors read and approved the final manuscript.

## Supplementary Material

Additional file 1 Some Cautions About Confounders, Multicollinearity, Suppression Effects, and Overadjustment in Linear Model Analyses. Click here for file

Additional file 2 Results of multiple linear regression analysis of association between natural log transformed total serum concentrations of persistent organic pollutants and lipid fractions (n = 575). Click here for file

Additional file 3 **Pearson’s correlation coefficients for natural log transformed serum concentrations of groups of PCBs based on the total number and number of *ortho *** **substituted chlorines and the various pesticides and pesticide groups.** Click here for file

Additional file 4 Pearson’s correlation coefficients for natural log transformed PCB congeners. Click here for file
