# Supplementary material for: Analysis of the effects of exposure to polychlorinated biphenyls and chlorinated pesticides on serum lipid levels in residents of Anniston, Alabama
Source: Environ Health. 2013 Dec 11;12:108. doi: 10.1186/1476-069X-12-108 (PMC3893492; doi:10.1186/1476-069X-12-108)
Supplement: Additional file 1 — Some Cautions About Confounders, Multicollinearity, Suppression Effects, and Overadjustment in Linear Model Analyses. [file 1476-069X-12-108-S1.docx]

**Additional file 1**

***Some Cautions About Confounders, Multicollinearity, Suppression Effects, and Overadjustment in Linear Model Analyses***

It is striking how different some of the results are when adjusted for other POPs. However we cannot eliminate the possibility that we are simply observing a statistical artifact of suppression or that some other lipophilic chemical that was not measured but migrates together with PCBs and chlorinated pesticides has the dominant action. It is well recognized that multicollinearity, the extent to which predictor variables in a multiple regression analysis share common variance, can have seriously debilitating effects on the analysis and its interpretation. As predictor variables share more and more variance in common, the individual regression coefficients ($\beta$) can become very unstable and standard errors of the parameter estimates and their related confidence intervals inflate concomitantly, which in turn results in a loss of power of statistical tests on the parameter estimates which diminishes proportionately (1-2). There is an even more insidious result of excessive collinearity that under the right circumstances can even reverse the sign of the original crude relationship (say, $r_{yx}$) and lead to statistically significant, but biologically implausible, interpretations. This condition—suppressor variable effects—is well known in statistics (3) and in the social sciences (4-8) and is becoming better known in medical and epidemiological applications (9). Because there are several instances of such suppression effects in the current study, it seemed prudent to discuss the issue and consider some options for avoiding potentially misleading interpretations in similar research.

The key to understanding the suppression effect and its deleterious consequences in multiple predictor linear models (including multiple regression and analysis of variance) is to examine the effects of multicollinearity on the parameter estimates of the model. Consider a linear model in which a continuously distributed outcome variable, *Y*, is regressed on two predictor variables, $X_{1}\mathrm{and} X_{2}$, with the assumption of normally distributed disturbances, $y=\beta_{0}+\beta_{1}x_{1}+\beta_{2}x_{2}+\epsilon$ (The problems are most easily illustrated by a two-predictor model, but the solution extends without loss of generality to models with any number of predictors. The problems are often exacerbated by adding correlated predictor variables to the model.). The partial regression coefficients of each regressor, adjusted for the remaining regressors in the model, are estimated by,

|  | $\beta_{1}=\frac{r_{yx_{1}}-r_{yx_{2}}r_{x_{1}x_{2}}}{1-r_{x_{1}x_{2}}^{2}}$ $\beta_{2}=\frac{r_{yx_{2}}-r_{yx_{1}}r_{x_{1}x_{2}}}{1-r_{x_{1}x_{2}}^{2}}$ | (1) |
| --- | --- | --- |

The degree of multicollinearity in any given problem is diagnosed by the quantity $1-r_{x_{1}x_{2}}^{2}$. If the predictors are nearly perfectly correlated (i.e., no unique information in either after controlling for the other) then $\beta_{1}$ and $\beta_{2}$ inflate drastically by virtue of a “shrinking” denominators of Equation 1. The so-called Variance Inflation Factor (VIF = $\frac{1}{1-r_{x_{1}x_{2}}^{2}}$ ) is an element of the standard error of the regression coefficients; hence as the collinearity indexed by $r_{x_{1}x_{2}}^{2}$ increases, the standard error increases concomitantly, the value of the *t*-test drops and statistical power is compromised. These are the essential elements of how multicollinearity diminishes the ability to test hypotheses on $\beta_{1}$ and $\beta_{2}$.

The more insidious effect of collinearity—suppression and/or enhancement—occurs when conditions arise that two (or more) predictors are more highly correlated with each other than one or the other (or both) predictors are correlated with the outcome variable. It is surprising how frequently this condition arises in practice. It is very common to have clusters of relatively highly correlated predictors that represent higher order constructs entered into models with very modest correlations to criterion variables. The typical predictor-criterion $r^{2}$ in the social sciences hovers around .05, while it is not uncommon to have predictor-predictor $r^{2}$ in the neighborhood of .50 and above (4).

Observe the numerator of the formulae in Equation (1) as $r_{yx_{1}}-r_{yx_{2}}r_{x_{1}x_{2}}$ and assume that the correlation between the criterion and the first predictor is $r_{yx_{1}}$ = .05, between the criterion and the second predictor is $r_{yx_{2}}$ = .20, and $r_{x_{1}x_{2}}= .95$ and hence, $\beta_{1}= -.14$. A major diagnostic sign of suppression occurs when a partial regression coefficient lies outside the range of 0 and the simple Pearson correlation.

The literature on suppression effects has experienced many incarnations over the past 7 decades spawning considerable lack of agreement among authors as to what constitutes suppression, enhancement, redundancy, and among suppression types what constitutes the differences a classical suppression, net suppression, reciprocal suppression, cooperative suppression, and so on. For our purposes it is sufficient to know that the interpretive difficulties that may arise are a function of excessive collinearity among predictors in conjunction with weaker individual validities of the predictor-criterion relationships. Recent overviews of the many definitions of suppressor phenomenon can be found in Beckstead (10), and Paulhus et al. (11). The same problems that plague research in the analysis of latent variables (structural equation modeling) are reviewed by Maassen and Bakker (12).

A positive bivariate correlation between a predictor and a criterion and a negative regression coefficient for the predictor when adjusted for other predictor variables, even though biologically implausible, is a clear sign of suppression. A suppressor is a variable that shares less variance with a criterion than another predictor and which removes from the second predictor variable the between-predictor variance that is irrelevant to the criterion. The classic example is an increase in the predictability ($R^{2}$) of WWII pilot training success that is correlated with a written test of spatial ability when a test of verbal ability, unrelated to success, is included in the regression model. The shared variance between written measures of spatial and verbal ability (i.e., reading ability and speed) is irrelevant to pilot success and is “removed” in the regression adjustment. Hence one observes an increase in the semi-partial correlation ($r_{y\left( x_{1} | x_{2} \right)}^{2}$, 13) between the criterion and the first predictor after adjustment for the suppressor predictor. The essential judgment that must be made in order to trust such an increase in $r_{y\left( x_{1} | x_{2} \right)}^{2}$ and/or $R_{y{.x}_{1}x_{2}}^{2}$due to a suppressor variable hinges critically on being able to identify the substantive construct that defines and explains the “irrelevant” variance shared between the predictor and the suppressor. Suppression effects are not limited to a single suppressor variable. The suppression effect may be composed of more complicated collections of additional predictors (14). In order for the suppressed effect of a predictor to be a genuine interpretable effect, there must be a substantive and theoretically plausible definition of the irrelevant variance such that the suppressed effect can be logically interpreted, even a biologically implausible shift in coefficient sign (i.e., $+ r_{yx} \to-\beta_{y{.x}_{1}{|x}_{2}}$).

Suppression effects are often argued to be more elusive and unreplicable statistical artifacts than they are genuine, interpretable replicable phenomenon (15). Conversely, it has been argued by some that there are actually more legitimate replicable suppressor effects than previously thought (11). Darlington (5), Horst (6), Paulhus et al. (11) and Cohen et al. (4) give examples of plausible suppressor effects.

Our own view is that absent a logical, theoretically justifiable definition of the common latent variable of the irrelevant variance, combined with an explanation of the biological, psychological, or physical process by which the suppression/enhancement take place, the wisest course of action is to remain skeptical that a genuine effect has been observed that is not a statistical artifact.

While it may appear that the example given above is extreme, this is precisely the situation that occurs in the analysis of many of the relationships between PCBs and lipids as seen in Tables 4, 5 and 7 of this study. Note the output from Table 4 of Model 1 and Model 2 for triglycerides. The regression coefficient for the log-transformed triglycerides regressed on mono-ortho PCBs in the Model 1 that adjusts only for the demographic confounders of age, age^2^, race, gender, BMI, alcohol, smoking, and exercise was estimated as $\beta_{ln(trig)\cdot demographics}$ = .08, *p* < .001. Hence, triglycerides are predicted to increase significantly by about .08 log-transformed points for each log-unit change in exposure to mono-ortho PCBs. Conversely, Model 2 analysis, which adjusts for the remaining PCB groups in addition to the demographic confounders, in Table 4 and which adjusts for the full set of 5 pesticides listed in Table 6, leads to a *negative* parameter estimate for log-transformed triglycerides of $\beta_{ln(trig)\cdot demo+pesticides}$ = -.16, which is statistically significant ($p= .022$). However it is biologically implausible that increasing the exposure to mono-ortho PCBs by one log-unit would lead to a -.16 log-unit reduction in triglycerides. Clearly the effect is a suppressor for other variables in the model, or is itself suppressed by excessive multicollinearity among the variables in Model 2. As noted above, there are many definitions of the types of suppression that can occur as a result of multicollinearity in the presence of validities that are smaller than the collinear relationships. Net suppression and collaborative suppression are terms that have been applied to more than one predictor variable that are jointly suppressing each other.

The problem is clearly identified in the zero-order Pearson correlations among the predictors (the full correlation matrix is presented in Additional files 2 and 3). The bivariate correlations among the PCB’s alone are excessively high: $r_{mono-di}= .94, r_{mono-tritetra}=.93, r_{di-tritetra}=.87$. More revealingly, the tolerances of these three variables when regressed on *all* of the 13 predictors in Model 2 are .06, .02 and .04 which indicates that the proportion of variance in each predictor that is redundant (i.e., confounded) with the remaining predictors is a whopping $R_{j.other}^{2}=.94, .98, \mathrm{and} ,.96$ for the mono-ortho, di-ortho, and tri/tetra-ortho chlorinated PCB variables. Such high correlations among predictors, along with relatively modest correlations between predictors and criterion variables (on the order of *r* = .02 to .24) are a near-certain recipe for suppression to occur—both positive and negative. Suppression effects can render an estimated regression coefficient much larger (but of the same sign) than the coefficient that obtains when the suppressor is not included in the model. This is a much more insidious form of suppression. Coefficients can be distorted far beyond any reasonable predictive expectation (and remain statistically significant), but are not so easily detected unless care is taken to compare the effect to a more crude estimate that does not involve the suppression relationships. The suppressor effect of tri/tetra-ortho PCBs on total lipids and on total cholesterol of Table 4 are examples.

The variables involved in a suppression relationship can be diagnosed by a variety of methods. Comparing parameter estimates of the predictor variables when estimated in the full model against the bivariate Pearson correlation coefficients is the most simple of procedures (4). Any coefficient that lies outside the range of [0, $r_{yx}$] is involved in some way in a suppression relationship. Examination of the tolerances and variance inflation factors provided by most computing programs can also help to isolate the offending variables. A more comprehensive method of assessing variables involved in suppression is based on a confirmatory factor analytic technique that separates the criterion relevant variance (CRV) and the criterion irrelevant variance (CIV) into two orthogonal factors (10; 16). As we noted above, placing trust in the substantive meaning of a variable involved in a suppression effect can only be justified if the CIV can be isolated and substantively identified—identifying and naming the CIV factor is a necessary requirement. A simplified example using the current data can clarify the issues.

A linear model of log-transformed triglycerides regressed on three PCBs clusters (mono-ortho, di-ortho, and tri/tetra-ortho chlorinated) and age (ignoring the intercept) yields the following fitted result with unstandardized coefficients:

$$\hat{y}=.08\left( mono \right)-.34\left( di \right)+.25\left( tritetra \right)+.001\left( age \right)$$

Mono-ortho and di-ortho coefficients are significantly different from zero at *p* = .003. Compare these coefficients to the four separate bivariate regression models (crude models) that yielded parameter estimates of .02, .01, .03 and .003 for mono*-*ortho, di-ortho*,* tri/tetra-ortho and age, respectively. By the definition of a suppressor variable each of the predictors is involved in a suppression effect in some way, acting as either suppressor or suppressed, or both. The most serious suppression effect is that for the di-ortho predictor which by itself is positive and non-significant. In the presence of the remaining PCBs and age, however, it migrates to a statistically significant (*p* = .003) *negative* effect—hard to ignore, but biologically implausible.

In order to identify and separate out the criterion relevant variance from the criterion irrelevant variance we fitted a two-factor confirmatory factor analytic model (17) to these two sources of variance as suggested by McFatter (16) and Beckstead (10). The factor model fitted to the data for CRV and CIV factors is illustrated in Figure 1 in which the criterion and all predictors are permitted to load on the CRV factor while only the predictors are allowed to load on the CIV factor. The squared factor loadings, the $\lambda^{2}$ of Figure 1, represent the proportion of variance of each variable that is attributable to CRV or CIV. The sum of the two squared factor loadings and the error variances for each indicator variable sum to 1.00 indicating that the variance of each variable is decomposed into these three sources, which are summarized in Table 8.

In an ideal sense, an indicator not involved in suppression would have an irrelevant variance squared value of $\lambda_{CIV}^{2}$ = .000. Compared to this gold standard it can be seen from Appendix Table 1 that all of the PCB clusters are involved in suppression with 88%, 95% and 80% of their variance being irrelevant to the criterion. At 36% the predictor variable age is less involved than the PCB variables in the criterion irrelevant variance. Given the positive and negative parameter estimates of the linear equation above, the problem results in both suppression and enhancement—effects that are both over- and under-estimated. We are unable to identify the central substantive construct that defines the latent variable of the irrelevant variance. The CIV factor is completely orthogonal to the relevant variance of this decomposition and therefore must be defined by elements that are not part of the lipid-to-PCB construct of CRV. Given our inability to identify the irrelevant variance factor, it may be best to seek one of several solutions to avoid or minimize misinterpretation of these effects in Model 2.

One solution to avoiding suppression and/or enhancement effects is to simply sum the PCB congeners into a single total PCB score, a tactic that is used in many studies of the health effects of PCBs (see for example, 18-19). Examination of Table 3 of the current study reveals no obvious suppressor effects when the data are treated in this fashion. A second viable solution would be to drop (or not include) any offending variables in the analyses as was done in the current study under the heading of Model 1 in the tabled regression results. These analyses adjust for the primary demographics in this domain, but do not include conceptually and statistically redundant predictor variables for which no causal model can be invoked (20-21).

Finally, an emerging method for handling excessive multicollinearity, and therefore suppression effects under the right circumstances, is the analysis of complex data such as these in structural equation models (22-23). Forming a latent variable of PCBs with measured indicators consisting of the congeners, or combinations of congeners, allows one to capitalize on the multicollinearity (correlations) among the indicators and at the same time model the unreliability of the measured variables. Consequently the regression models on latent variables (17) are disattenuated, are most likely stronger predictors, and are free of the difficulties of multicollinearity, suppression and enhancement. For an example of such a strategy in the PCB domain see Goncharev et al. [24]. No matter which strategy is employed to address the problems of suppression, we raise the issue here to alert investigators to a difficulty that will almost certainly arise in studies with many predictors which are often taken from systemically correlated domains.

We might end the cautionary note of this section with one more example from the current data of yet an additional complication that can arise in complex research problems based on observational data. The crude bivariate effect of log-transformed total PCBs on log-transformed triglycerides ($\hat{\beta}=.019, p= .317at a al Pesticides are after adjusting for the demographic covariates is only marginally reduced when lems for which there$) is positive, as is the crude bivariate effect of log-transformed total pesticides ($\hat{\beta}=.069, p= .003at a al Pesticides are after adjusting for the demographic covariates is only marginally reduced when lems for which there$). Estimating the model with PCBs and pesticides together, however, produces a statistically significant reversal of sign for the PCBs with the fitted model parameter estimates and *p*-values for PCBs and pesticides, respectively, being $\hat{\beta}_{PCBs}=-.056, p=.028$ and $\hat{\beta}_{Pest}=.122, p<.001$. The effects of suppression and enhancement are apparent. It turns out, however, that the suppression effect in this example is directly related to an underlying case of Simpson’s Paradox (25) in which the regression slopes within strata can be of one direction while the direction of the effect estimated on the whole sample can be opposite that of the stratified estimates. Tu Gunnell and Gilthorpe (26) include Simpson’s paradox as an element of the class of confounders accompanied by the suppression effect. Simpson’s paradox, in this instance revolves around the race of the respondents (Caucasian and African American). A graphical illustration of the underlying paradox is shown in Figures 2, 3a and 3b. Because the distributions of African American and Caucasian respondents for triglycerides and for PCB exposure are non-overlapping in opposite directions (Figure 2), the positive fit of the individual bivariate slopes (Figure 3a) are overridden and masked when the fit of the model is based on the combined samples (Figure 3b). These differences by race are presented in detail in a subsequent publication (27). When race is added to the regression of triglycerides on both PCBs and pesticides, the negative sign for PCBs disappears, the PCB effect is no longer statistically significant, and the pesticides adjusted for race and PCBs retain their significant positive effect on log-transformed triglycerides. It is worth noting that what appears as a case of suppression in the analysis is due to conditions required for suppression which can in turn be augmented by the reversal effect of Simpson’s paradox (9,26). Because race of the respondent is one of the demographic covariates adjusted for in these analyses, the paradox is benign and therefore not evident in the results of Table 3.

The situation of appropriately adjusting for potential confounds is even more complicated than often thought. The design advice offered by the application of directed acylclic graphs (DAGs) to provide visual assistance to the logical process of selecting and controlling confounds in health research (see for example 28-31) is a useful tool but even skillfully applied can fail to identify all the difficulties that can arise in complex studies (31). Finally, attempting to be inclusive by specifying too many covariates can easily lead to overadjustment and serious model misspecifications, similar to those encountered in the present data (32).

Bibliography:

1. Draper NR, Smith H. *Applied Regression Analysis.* New York: Wiley; 1998.
2. Fox J: *Applied Regression Analysis and Generalized Linear Models.* Thousand Oaks, CA: Sage Publications; 2008.
3. Lewis JW, Escobar LA: **Suppression and enhancement in bivariate regression.** *The Statistician* 1986, **35:**17-26.
4. Cohen J, Cohen P, West SG, Aiken LS: *Applied Multiple Correlation/Regression Analysis for the Behavioral Sciences.*  Mahwah, NJ: Lawrence Erlbaum Associates Publishers; 2003.
5. Darlington RD: *Regression and Linear Models.* New York: McGraw-Hill; 1990.
6. Horst P: **The prediction of personal adjustment.** *Soc Sci Res Bul* 1941, **48:**431-436.
7. Smith RL, Ager JW, Williams DL: **Suppressor variables in multiple regression/correlation*.*** *Educ Psychol Meas* 1992, **52:**17-29.
8. Tzelgov J, Henik A: **Suppression situations in psychological research: Definitions, implications, and applications.** *Psychol Bul*, 1991, **109:**534-536.
9. Tu YK, West R, Ellison GTH, Gilthorpe MS: **Why evidence for the fetal origins of adult disease might be a statistical artifact: The “refersal paradox” for the relations between birth weight and blood pressure in later life**. *Am J Epidemiol* 2005, **161:**27-32.
10. Beckstead JW: **Isolating and examining sources of suppression and multicollinearity in multiple linear regression.** *Multivar Behav Res* 2012, **47:**224-246.
11. Paulhus DL, Robins RW, Trzesniewski KH, Tracy JL: **Two replicable suppressor situations in personality research.**  *Multivar Behav Res* 2004, **39:**303-328.
12. Maassen GH, Bakker AB: **Suppressor variables in path models.** *Sociol Meth Res* 2001, **30**:241-270.
13. Velicer WF: **Suppressor variables and the semipartial correlation coefficient.** *Educ Psychol Meas* 1978, **38:**953-958.
14. Holling H: **Suppressor structures in the general linear model.** *Educ Psychol Meas* 1983, **43**:1-9
15. Wiggins JS: *Personality and Prediction: Principles of Personality Assessment*. Reading, MA: Addison-Wesley, 1973.
16. McFatter RM**: The use of structural equation models in interpreting regression equations including suppressor variables and enhancer variables.** *Appl Psych Meas* 1979, **3:**123-135.
17. Bollen KA: *Structural Equations With Latent Variables.* New York: Wiley; 1989.
18. Schantz SL, Gasior DM, Polverejan E, McCaffrey RJ, Sweeney AM, Humphrey HE, Gardiner JC: **Impairments of memory and learning in older adults exposed to polychlorinated biphenyls via consumption of Great Lakes fish.** *Environ Health Perspect* 2001, **109:**605-611.
19. Gustavsson P, Hogstedt C**: A cohort study of Swedish capacitor manufacturing workers exposed to polychlorinated biphenyls (PCBs).** *Am J Ind Med* 1997, **32:**234-239.
20. Shipley B*. Cause and Correlation in Biology.* Cambridge, UK: Cambridge University Press; 2002.
21. Hernán MA: **A definition of causal effect for epidemiological research.** *J Epidemiol Community Health* 2004, **58:**265-271.
22. Muthen BO: **Latent variable modeling in epidemiology.** *Alcohol Health Res W* 1992, **16:**286-292.
23. Tu YK: **Commentary: Is structural equation modeling a step forward for epidemiologists?** *Int J Epidemiol* 2009, **38:**549-551.
24. Goncharov A, Haase RF, Santiago-Rivera A, Morse G; Akwesasne Task Force on the Environment, McCaffrey RJ, Rej R, Carpenter DO: **High serum PCBs are associated with elevation of serum lipids and cardiovascular disease in a Native American population.** Environ Res 2008, **106:**226-239.
25. Simpson, EH: **The interpretation of interaction in contingency tables**. *J. R. Stat Soc B* 1951, **2**:238-241.
26. Tu YK, Gunnell D, Gilthrope MS: **Simpson’s paradox, Lord’s paradox, and suppression effects are the same phenomenon—the reversal paradox.** *Emerging Themes Epidemiol* 2008, **5:**1-9.
27. Aminov Z, Haase R, Olson JR, Pavuk M, Carpenter DO: **Racial Differences in Levels of Serum Lipids and Effects of Exposure to Persistent Organic Pollutants on Lipid Levels in Residents of Anniston, Alabama.** 2013, Submitted for publication.
28. Pearl J. Causality: *Modeling, Reasoning and Inference*. Cambridge, UK: Cambridge University Press, 2000.
29. Greenland S, Pearl J, Robins JM: **Causal diagrams for epidemiologic research**. *Epidemiology* 1999, **10**:37-48.
30. Glymour M, Greenland, S: **Causal diagrams**. In *Modern Epidemiology*. Edited by Rothman KJ, Greenland, S, Lash TL. Philadelphia: Lippencott, Williams & Wilkens; 2008.
31. Hernán MA, Hernández-Díaz S, Werler MM, Mitchell AA: **Causal knowledge as a prerequisite for confounding evaluation: An application to birth defects epidemiology.** *Am J Epidemiol* 2002, **155:**176-184.
32. Hernández-Díaz S, Schisterman EF, Hernán MA: **The birth weight “paradox” uncovered?** *Am J Epidemiol* 2006, **164**:1124-1125.
33. Schisterman EF, Cole SR, Platt RW: **Overadjustment bias and unnecessary adjustment in epidemiologic studies**. *Epidemiology* 2009, **20**:488-495.

Appendix Table 1. Decomposition of the Criterion Relevant ($\lambda_{CRV}^{2}$) and Criterion Irrelevant ($\lambda_{CIV}^{2}$) Variance

| Variable | $\lambda_{CRV}^{2}$ | $\lambda_{CIV}^{2}$ | $\Theta_{\delta}$ |
| --- | --- | --- | --- |
| total lipids | .058 | --- | --- |
| mono-ortho | .557 | .444 | .000 |
| di-ortho | .348 | .567 | .084 |
| tri/tetra-ortho | .645 | .315 | .040 |
| Age | .612 | .111 | .278 |

Figure 1. Confirmatory Factor Model of Relevant and Irrelevant Variance for the relationships among serum triglycerides with groups PCBs and age. CRV = criteria relevant variance. CIV = criteria irrelevant variance.

Figure 2. Simpson’s Paradox: Oppositely Overlapping Histograms of Triglycerides and Pesticides by Race


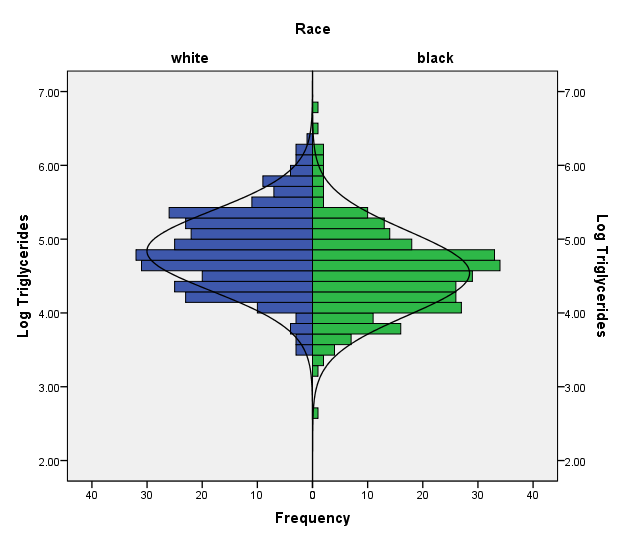

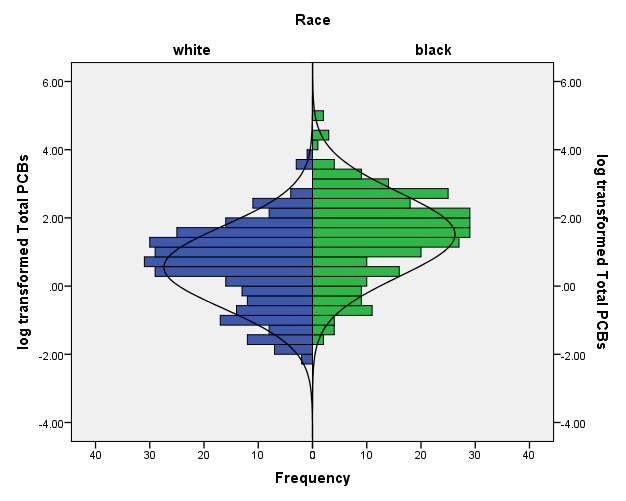


Figure 3. Simpson’s Paradox: Scatterplots of the Triglyceride-on-PCB Relationship by Race and for the Whole Sample

(a)

Note: Slopes within strata (red lines) are positive and significant (white .072, p = .007 and

black .069, p = .012); the slope for the whole sample ignoring race (green line) is nearer zero,

positive and not significant (.019, p = .327 ).

(b)

Note: Slope for PCBs adjusted for race is .070, p < .001.
