# Supplementary material for: Analysis of the effects of exposure to polychlorinated biphenyls and chlorinated pesticides on serum lipid levels in residents of Anniston, Alabama
Source: Environ Health. 2013 Dec 11;12:108. doi: 10.1186/1476-069X-12-108 (PMC3893492; doi:10.1186/1476-069X-12-108)
Supplement: Additional file 2 — Results of multiple linear regression analysis of association between natural log transformed total serum concentrations of persistent organic pollutants and lipid fractions (n = 575). [file 1476-069X-12-108-S2.docx]

Additional file 2. Results of multiple linear regression analysis of association between natural log transformed total serum concentrations of persistent organic pollutants and lipid fractions (n=575).

|  | | Total POPs |
| --- | --- | --- |
| Total lipids | β | 0.07 |
|  | SE | 0.01 |
|  | p-value | <0.0001* |
|  | SP R^2^2 | 0.0401 |
| Total Cholesterol | β | 0.04 |
|  | SE | 0.01 |
|  | p-value | 0.0035* |
|  | SP R^2^2 | 0.0139 |
| HDL cholesterol | β | -0.01 |
|  | SE | 0.02 |
|  | p-value | 0.7345 |
|  | SP R^2^2 | 0.0002 |
| LDL cholesterol | β | 0.03 |
|  | SE | 0.02 |
|  | p-value | 0.1292 |
|  | SP R^2^2 | 0.0039 |
| Triglycerides | β | 0.15 |
|  | SE | 0.03 |
|  | p-value | <0.0001* |
|  | SP R^2^2 | 0.0292 |

The estimates are adjusted for age (as well as age quadratic), race, gender, BMI, alcohol consumption, smoking and exercising status.

*Results were considered significant at p<0.05.

SP R^2^2 – Type II semi-partial R^2^.
