# Supplementary material for: Analysis of the effects of exposure to polychlorinated biphenyls and chlorinated pesticides on serum lipid levels in residents of Anniston, Alabama
Source: Environ Health. 2013 Dec 11;12:108. doi: 10.1186/1476-069X-12-108 (PMC3893492; doi:10.1186/1476-069X-12-108)
Supplement: Additional file 3 — Pearson’s correlation coefficients for natural log transformed serum concentrations of groups of PCBs based on the total number and number of ortho substituted chlorines and the various pesticides and pesticide groups. [file 1476-069X-12-108-S3.docx]

Additional file 3. Pearson’s correlation coefficients for natural log transformed serum concentrations of groups of PCBs based on the total number and number of *ortho* substituted chlorines and the various pesticides and pesticide groups.

|  | Total POPs | Total PCB | Total pesticides | Mono-ortho PCBs | Di-ortho PCBs | Tri/Tetra-ortho PCBs | Tri/ Tetrachloro PCBs | Pentachloro PCBs | Hexachloro PCBs | Heptachloro PCBs | Octa/Nona/ Decachloro PCBs | DDT | Chlordane | Mirex | HCB | HCCH |
| --- | --- | --- | --- | --- | --- | --- | --- | --- | --- | --- | --- | --- | --- | --- | --- | --- |
| Total POPs | 1 | 0.95 | 0.91 | 0.92 | 0.94 | 0.92 | 0.87 | 0.88 | 0.94 | 0.93 | 0.90 | 0.88 | 0.76 | 0.77 | 0.63 | 0.74 |
|  |  | <0.0001 | <0.0001 | <0.0001 | <0.0001 | <0.0001 | <0.0001 | <0.0001 | <0.0001 | <0.0001 | <0.0001 | <0.0001 | <0.0001 | <0.0001 | <0.0001 | <0.0001 |
|  | 573 | 573 | 573 | 573 | 573 | 573 | 573 | 573 | 573 | 573 | 573 | 571 | 570 | 573 | 573 | 570 |
| Total PCB |  | 1 | 0.76 | 0.95 | 1.00 | 0.98 | 0.86 | 0.90 | 0.99 | 0.99 | 0.96 | 0.72 | 0.69 | 0.80 | 0.53 | 0.64 |
|  |  |  | <0.0001 | <0.0001 | <0.0001 | <0.0001 | <0.0001 | <0.0001 | <0.0001 | <0.0001 | <0.0001 | <0.0001 | <0.0001 | <0.0001 | <0.0001 | <0.0001 |
|  |  | 573 | 573 | 573 | 573 | 573 | 573 | 573 | 573 | 573 | 573 | 571 | 570 | 573 | 573 | 570 |
| Total pesticides |  |  | 1 | 0.78 | 0.76 | 0.73 | 0.79 | 0.75 | 0.77 | 0.74 | 0.71 | 0.98 | 0.79 | 0.63 | 0.70 | 0.80 |
|  |  |  |  | <0.0001 | <0.0001 | <0.0001 | <0.0001 | <0.0001 | <0.0001 | <0.0001 | <0.0001 | <0.0001 | <0.0001 | <0.0001 | <0.0001 | <0.0001 |
|  |  |  | 573 | 573 | 573 | 573 | 573 | 573 | 573 | 573 | 573 | 571 | 570 | 573 | 573 | 570 |
| Mono-ortho PCBs |  |  |  | 1 | 0.94 | 0.89 | 0.95 | 0.97 | 0.95 | 0.92 | 0.85 | 0.75 | 0.69 | 0.71 | 0.60 | 0.69 |
|  |  |  |  |  | <0.0001 | <0.0001 | <0.0001 | <0.0001 | <0.0001 | <0.0001 | <0.0001 | <0.0001 | <0.0001 | <0.0001 | <0.0001 | <0.0001 |
|  |  |  |  | 573 | 573 | 573 | 573 | 573 | 573 | 573 | 573 | 571 | 570 | 573 | 573 | 570 |
| Di-ortho PCBs |  |  |  |  | 1 | 0.97 | 0.84 | 0.90 | 0.99 | 0.99 | 0.94 | 0.72 | 0.68 | 0.80 | 0.53 | 0.62 |
|  |  |  |  |  |  | <0.0001 | <0.0001 | <0.0001 | <0.0001 | <0.0001 | <0.0001 | <0.0001 | <0.0001 | <0.0001 | <0.0001 | <0.0001 |
|  |  |  |  |  | 573 | 573 | 573 | 573 | 573 | 573 | 573 | 571 | 570 | 573 | 573 | 570 |
| Tri/Tetra-ortho PCBs |  |  |  |  |  | 1 | 0.80 | 0.83 | 0.95 | 0.98 | 0.99 | 0.68 | 0.69 | 0.81 | 0.51 | 0.62 |
|  |  |  |  |  |  |  | <0.0001 | <0.0001 | <0.0001 | <0.0001 | <0.0001 | <0.0001 | <0.0001 | <0.0001 | <0.0001 | <0.0001 |
|  |  |  |  |  |  | 573 | 573 | 573 | 573 | 573 | 573 | 571 | 570 | 573 | 573 | 570 |
| Tri/ Tetrachloro PCBs |  |  |  |  |  |  | 1 | 0.90 | 0.86 | 0.82 | 0.77 | 0.76 | 0.72 | 0.62 | 0.66 | 0.75 |
|  |  |  |  |  |  |  |  | <0.0001 | <0.0001 | <0.0001 | <0.0001 | <0.0001 | <0.0001 | <0.0001 | <0.0001 | <0.0001 |
|  |  |  |  |  |  |  | 573 | 573 | 573 | 573 | 573 | 571 | 570 | 573 | 573 | 570 |
| Pentachloro PCBs |  |  |  |  |  |  |  | 1 | 0.93 | 0.85 | 0.76 | 0.75 | 0.60 | 0.62 | 0.55 | 0.63 |
|  |  |  |  |  |  |  |  |  | <0.0001 | <0.0001 | <0.0001 | <0.0001 | <0.0001 | <0.0001 | <0.0001 | <0.0001 |
|  |  |  |  |  |  |  |  | 573 | 573 | 573 | 573 | 571 | 570 | 573 | 573 | 570 |
| Hexachloro PCBs |  |  |  |  |  |  |  |  | 1 | 0.98 | 0.91 | 0.75 | 0.67 | 0.78 | 0.53 | 0.62 |
|  |  |  |  |  |  |  |  |  |  | <0.0001 | <0.0001 | <0.0001 | <0.0001 | <0.0001 | <0.0001 | <0.0001 |
|  |  |  |  |  |  |  |  |  | 573 | 573 | 573 | 571 | 570 | 573 | 573 | 570 |
| Heptachloro PCBs |  |  |  |  |  |  |  |  |  | 1 | 0.96 | 0.70 | 0.68 | 0.83 | 0.51 | 0.61 |
|  |  |  |  |  |  |  |  |  |  |  | <0.0001 | <0.0001 | <0.0001 | <0.0001 | <0.0001 | <0.0001 |
|  |  |  |  |  |  |  |  |  |  | 573 | 573 | 571 | 570 | 573 | 573 | 570 |
| Octa/Nona/ Decachloro PCBs |  |  |  |  |  |  |  |  |  |  | 1 | 0.65 | 0.70 | 0.81 | 0.50 | 0.62 |
|  |  |  |  |  |  |  |  |  |  |  |  | <0.0001 | <0.0001 | <0.0001 | <0.0001 | <0.0001 |
|  |  |  |  |  |  |  |  |  |  |  | 573 | 571 | 570 | 573 | 573 | 570 |
| DDT |  |  |  |  |  |  |  |  |  |  |  | 1 | 0.70 | 0.56 | 0.65 | 0.75 |
|  |  |  |  |  |  |  |  |  |  |  |  |  | <0.0001 | <0.0001 | <0.0001 | <0.0001 |
|  |  |  |  |  |  |  |  |  |  |  |  | 571 | 568 | 571 | 571 | 568 |
| Chlordane |  |  |  |  |  |  |  |  |  |  |  |  | 1 | 0.65 | 0.68 | 0.77 |
|  |  |  |  |  |  |  |  |  |  |  |  |  |  | <0.0001 | <0.0001 | <0.0001 |
|  |  |  |  |  |  |  |  |  |  |  |  |  | 570 | 570 | 570 | 567 |
| Mirex |  |  |  |  |  |  |  |  |  |  |  |  |  | 1 | 0.40 | 0.49 |
|  |  |  |  |  |  |  |  |  |  |  |  |  |  |  | <0.0001 | <0.0001 |
|  |  |  |  |  |  |  |  |  |  |  |  |  |  | 573 | 573 | 570 |
| HCB |  |  |  |  |  |  |  |  |  |  |  |  |  |  | 1 | 0.76 |
|  |  |  |  |  |  |  |  |  |  |  |  |  |  |  |  | <0.0001 |
|  |  |  |  |  |  |  |  |  |  |  |  |  |  |  | 573 | 570 |
| HCCH |  |  |  |  |  |  |  |  |  |  |  |  |  |  |  | 1 |
|  |  |  |  |  |  |  |  |  |  |  |  |  |  |  |  |  |
|  |  |  |  |  |  |  |  |  |  |  |  |  |  |  |  | 570 |
