# Supplementary material for: Analysis of the effects of exposure to polychlorinated biphenyls and chlorinated pesticides on serum lipid levels in residents of Anniston, Alabama
Source: Environ Health. 2013 Dec 11;12:108. doi: 10.1186/1476-069X-12-108 (PMC3893492; doi:10.1186/1476-069X-12-108)
Supplement: Additional file 4 — Pearson’s correlation coefficients for natural log transformed PCB congeners. [file 1476-069X-12-108-S4.docx]

Additional file 4. Pearson’s correlation coefficients for natural log transformed PCB congeners.

|  | 28 | 66 | 74 | 118 | 157 | 167 | 189 | 87 | 99 | 105 | 146 | 153 | 156 | 138-158 | 170 | 172 | 180 | 194 | 177 | 178 | 183 | 187 | 195 | 199 | 196-203 | 206 | 209 |
| --- | --- | --- | --- | --- | --- | --- | --- | --- | --- | --- | --- | --- | --- | --- | --- | --- | --- | --- | --- | --- | --- | --- | --- | --- | --- | --- | --- |
| **28** | 1    573 | 0.80  <.0001  573 | 0.61  <.0001  573 | 0.57  <.0001  573 | 0.42  <.0001  573 | 0.49  <.0001  573 | 0.37  <.0001  573 | 0.41  <.0001  558 | 0.45  <.0001  573 | 0.59  <.0001  551 | 0.39  <.0001  573 | 0.42  <.0001  573 | 0.41  <.0001  573 | 0.44  <.0001  573 | 0.40  <.0001  573 | 0.39  <.0001  573 | 0.40  <.0001  573 | 0.37  <.0001  570 | 0.42  <.0001  573 | 0.36  <.0001  573 | 0.43  <.0001  573 | 0.39  <.0001  573 | 0.42  <.0001  570 | 0.39  <.0001  573 | 0.41  <.0001  573 | 0.41  <.0001  572 | 0.39  <.0001  573 |
| **66** |  | 1    573 | 0.74  <.0001  573 | 0.81  <.0001  573 | 0.62  <.0001  573 | 0.72  <.0001  573 | 0.58  <.0001  573 | 0.59  <.0001  558 | 0.73  <.0001  573 | 0.85  <.0001  551 | 0.65  <.0001  573 | 0.66  <.0001  573 | 0.59  <.0001  573 | 0.68  <.0001  573 | 0.60  <.0001  573 | 0.61  <.0001  573 | 0.58  <.0001  573 | 0.52  <.0001  570 | 0.68  <.0001  573 | 0.59  <.0001  573 | 0.67  <.0001  573 | 0.64  <.0001  573 | 0.62  <.0001  570 | 0.54  <.0001  573 | 0.58  <.0001  573 | 0.52  <.0001  572 | 0.47  <.0001  573 |
| **74** |  |  | 1    573 | 0.92  <.0001  573 | 0.89  <.0001  573 | 0.91  <.0001  573 | 0.78  <.0001  573 | 0.58  <.0001  558 | 0.85  <.0001  573 | 0.88  <.0001  551 | 0.84  <.0001  573 | 0.89  <.0001  573 | 0.89  <.0001  573 | 0.89  <.0001  573 | 0.87  <.0001  573 | 0.84  <.0001  573 | 0.86  <.0001  573 | 0.79  <.0001  570 | 0.88  <.0001  573 | 0.82  <.0001  573 | 0.87  <.0001  573 | 0.85  <.0001  573 | 0.85  <.0001  570 | 0.81  <.0001  573 | 0.84  <.0001  573 | 0.80  <.0001  572 | 0.76  <.0001  573 |
| **118** |  |  |  | 1    573 | 0.85  <.0001  573 | 0.94  <.0001  573 | 0.75  <.0001  573 | 0.64  <.0001  558 | 0.92  <.0001  573 | 0.98  <.0001  551 | 0.87  <.0001  573 | 0.89  <.0001  573 | 0.83  <.0001  573 | 0.90  <.0001  573 | 0.82  <.0001  573 | 0.81  <.0001  573 | 0.81  <.0001  573 | 0.72  <.0001  570 | 0.90  <.0001  573 | 0.80  <.0001  573 | 0.89  <.0001  573 | 0.85  <.0001  573 | 0.83  <.0001  570 | 0.75  <.0001  573 | 0.78  <.0001  573 | 0.73  <.0001  572 | 0.66  <.0001  573 |
| **157** |  |  |  |  | 1    573 | 0.93  <.0001  573 | 0.92  <.0001  573 | 0.54  <.0001  558 | 0.83  <.0001  573 | 0.80  <.0001  551 | 0.93  <.0001  573 | 0.95  <.0001  573 | 0.98  <.0001  573 | 0.93  <.0001  573 | 0.97  <.0001  573 | 0.95  <.0001  573 | 0.96  <.0001  573 | 0.92  <.0001  570 | 0.92  <.0001  573 | 0.94  <.0001  573 | 0.91  <.0001  573 | 0.94  <.0001  573 | 0.95  <.0001  570 | 0.93  <.0001  573 | 0.94  <.0001  573 | 0.90  <.0001  572 | 0.85  <.0001  573 |
| **167** |  |  |  |  |  | 1    573 | 0.85  <.0001  573 | 0.60  <.0001  558 | 0.89  <.0001  573 | 0.90  <.0001  551 | 0.92  <.0001  573 | 0.94  <.0001  573 | 0.91  <.0001  573 | 0.93  <.0001  573 | 0.90  <.0001  573 | 0.90  <.0001  573 | 0.90  <.0001  573 | 0.83  <.0001  570 | 0.93  <.0001  573 | 0.89  <.0001  573 | 0.91  <.0001  573 | 0.92  <.0001  573 | 0.90  <.0001  570 | 0.85  <.0001  573 | 0.87  <.0001  573 | 0.81  <.0001  572 | 0.76  <.0001  573 |
| **189** |  |  |  |  |  |  | 1    573 | 0.50  <.0001  558 | 0.75  <.0001  573 | 0.71  <.0001  551 | 0.86  <.0001  573 | 0.87  <.0001  573 | 0.89  <.0001  573 | 0.85  <.0001  573 | 0.91  <.0001  573 | 0.92  <.0001  573 | 0.90  <.0001  573 | 0.88  <.0001  570 | 0.86  <.0001  573 | 0.89  <.0001  573 | 0.84  <.0001  573 | 0.88  <.0001  573 | 0.91  <.0001  570 | 0.89  <.0001  573 | 0.89  <.0001  573 | 0.87  <.0001  572 | 0.83  <.0001  573 |
| **87** |  |  |  |  |  |  |  | 1    558 | 0.69  <.0001  558 | 0.66  <.0001  536 | 0.58  <.0001  558 | 0.60  <.0001  558 | 0.51  <.0001  558 | 0.63  <.0001  558 | 0.52  <.0001  558 | 0.53  <.0001  558 | 0.50  <.0001  558 | 0.44  <.0001  555 | 0.61  <.0001  558 | 0.50  <.0001  558 | 0.62  <.0001  558 | 0.55  <.0001  558 | 0.54  <.0001  555 | 0.46  <.0001  558 | 0.50  <.0001  558 | 0.43  <.0001  557 | 0.39  <.0001  558 |
| **99** |  |  |  |  |  |  |  |  | 1    573 | 0.92  <.0001  551 | 0.89  <.0001  573 | 0.92  <.0001  573 | 0.82  <.0001  573 | 0.94  <.0001  573 | 0.83  <.0001  573 | 0.81  <.0001  573 | 0.81  <.0001  573 | 0.72  <.0001  570 | 0.94  <.0001  573 | 0.81  <.0001  573 | 0.94  <.0001  573 | 0.88  <.0001  573 | 0.84  <.0001  570 | 0.75  <.0001  573 | 0.80  <.0001  573 | 0.71  <.0001  572 | 0.64  <.0001  573 |
| **105** |  |  |  |  |  |  |  |  |  | 1    551 | 0.83  <.0001  551 | 0.86  <.0001  551 | 0.78  <.0001  551 | 0.87  <.0001  551 | 0.77  <.0001  551 | 0.77  <.0001  551 | 0.75  <.0001  551 | 0.67  <.0001  548 | 0.88  <.0001  551 | 0.75  <.0001  551 | 0.86  <.0001  551 | 0.82  <.0001  551 | 0.79  <.0001  548 | 0.70  <.0001  551 | 0.74  <.0001  551 | 0.67  <.0001  550 | 0.61  <.0001  551 |
| **146** |  |  |  |  |  |  |  |  |  |  | 1    573 | 0.97  <.0001  573 | 0.93  <.0001  573 | 0.95  <.0001  573 | 0.94  <.0001  573 | 0.92  <.0001  573 | 0.93  <.0001  573 | 0.88  <.0001  570 | 0.95  <.0001  573 | 0.93  <.0001  573 | 0.94  <.0001  573 | 0.96  <.0001  573 | 0.92  <.0001  570 | 0.89  <.0001  573 | 0.91  <.0001  573 | 0.84  <.0001  572 | 0.78  <.0001  573 |
| **153** |  |  |  |  |  |  |  |  |  |  |  | 1    573 | 0.95  <.0001  573 | 0.98  <.0001  573 | 0.97  <.0001  573 | 0.94  <.0001  573 | 0.96  <.0001  573 | 0.89  <.0001  570 | 0.98  <.0001  573 | 0.94  <.0001  573 | 0.97  <.0001  573 | 0.98  <.0001  573 | 0.95  <.0001  570 | 0.91  <.0001  573 | 0.94  <.0001  573 | 0.86  <.0001  572 | 0.80  <.0001  573 |
| **156** |  |  |  |  |  |  |  |  |  |  |  |  | 1    573 | 0.93  <.0001  573 | 0.98  <.0001  573 | 0.95  <.0001  573 | 0.98  <.0001  573 | 0.94  <.0001  570 | 0.92  <.0001  573 | 0.95  <.0001  573 | 0.92  <.0001  573 | 0.95  <.0001  573 | 0.94  <.0001  570 | 0.95  <.0001  573 | 0.95  <.0001  573 | 0.91  <.0001  572 | 0.86  <.0001  573 |
| **138-158** |  |  |  |  |  |  |  |  |  |  |  |  |  | 1    573 | 0.94  <.0001  573 | 0.92  <.0001  573 | 0.93  <.0001  573 | 0.86  <.0001  570 | 0.97  <.0001  573 | 0.91  <.0001  573 | 0.96  <.0001  573 | 0.95  <.0001  573 | 0.92  <.0001  570 | 0.88  <.0001  573 | 0.91  <.0001  573 | 0.83  <.0001  572 | 0.77  <.0001  573 |
| **170** |  |  |  |  |  |  |  |  |  |  |  |  |  |  | 1    573 | 0.97  <.0001  573 | 1.00  <.0001  573 | 0.96  <.0001  570 | 0.93  <.0001  573 | 0.96  <.0001  573 | 0.93  <.0001  573 | 0.97  <.0001  573 | 0.96  <.0001  570 | 0.97  <.0001  573 | 0.98  <.0001  573 | 0.93  <.0001  572 | 0.87  <.0001  573 |
| **172** |  |  |  |  |  |  |  |  |  |  |  |  |  |  |  | 1    573 | 0.97  <.0001  573 | 0.94  <.0001  570 | 0.92  <.0001  573 | 0.95  <.0001  573 | 0.91  <.0001  573 | 0.95  <.0001  573 | 0.95  <.0001  570 | 0.94  <.0001  573 | 0.95  <.0001  573 | 0.90  <.0001  572 | 0.85  <.0001  573 |
| **180** |  |  |  |  |  |  |  |  |  |  |  |  |  |  |  |  | 1    573 | 0.96  <.0001  570 | 0.92  <.0001  573 | 0.96  <.0001  573 | 0.92  <.0001  573 | 0.97  <.0001  573 | 0.96  <.0001  570 | 0.97  <.0001  573 | 0.98  <.0001  573 | 0.93  <.0001  572 | 0.88  <.0001  573 |
| **194** |  |  |  |  |  |  |  |  |  |  |  |  |  |  |  |  |  | 1    570 | 0.85  <.0001  570 | 0.93  <.0001  570 | 0.85  <.0001  570 | 0.91  <.0001  570 | 0.92  <.0001  570 | 0.97  <.0001  570 | 0.96  <.0001  570 | 0.94  <.0001  569 | 0.89  <.0001  570 |
| **177** |  |  |  |  |  |  |  |  |  |  |  |  |  |  |  |  |  |  | 1    573 | 0.92  <.0001  573 | 0.97  <.0001  573 | 0.97  <.0001  573 | 0.94  <.0001  570 | 0.88  <.0001  573 | 0.91  <.0001  573 | 0.83  <.0001  572 | 0.76  <.0001  573 |
| **178** |  |  |  |  |  |  |  |  |  |  |  |  |  |  |  |  |  |  |  | 1    573 | 0.91  <.0001  573 | 0.95  <.0001  573 | 0.93  <.0001  570 | 0.93  <.0001  573 | 0.94  <.0001  573 | 0.88  <.0001  572 | 0.83  <.0001  573 |
| **183** |  |  |  |  |  |  |  |  |  |  |  |  |  |  |  |  |  |  |  |  | 1    573 | 0.95  <.0001  573 | 0.93  <.0001  570 | 0.88  <.0001  573 | 0.91  <.0001  573 | 0.83  <.0001  572 | 0.77  <.0001  573 |
| **187** |  |  |  |  |  |  |  |  |  |  |  |  |  |  |  |  |  |  |  |  |  | 1    573 | 0.95  <.0001  570 | 0.94  <.0001  573 | 0.95  <.0001  573 | 0.88  <.0001  572 | 0.82  <.0001  573 |
| **195** |  |  |  |  |  |  |  |  |  |  |  |  |  |  |  |  |  |  |  |  |  |  | 1    570 | 0.94  <.0001  570 | 0.95  <.0001  570 | 0.91  <.0001  569 | 0.86  <.0001  570 |
| **199** |  |  |  |  |  |  |  |  |  |  |  |  |  |  |  |  |  |  |  |  |  |  |  | 1    573 | 0.97  <.0001  573 | 0.96  <.0001  572 | 0.92  <.0001  573 |
| **196-203** |  |  |  |  |  |  |  |  |  |  |  |  |  |  |  |  |  |  |  |  |  |  |  |  | 1    573 | 0.95  <.0001  572 | 0.90  <.0001  573 |
| **206** |  |  |  |  |  |  |  |  |  |  |  |  |  |  |  |  |  |  |  |  |  |  |  |  |  | 1    572 | 0.98  <.0001  572 |
| **209** |  |  |  |  |  |  |  |  |  |  |  |  |  |  |  |  |  |  |  |  |  |  |  |  |  |  | 1    573 |
